# Bisphenol S rapidly depresses heart function through estrogen receptor-β and decreases phospholamban phosphorylation in a sex-dependent manner

**DOI:** 10.1038/s41598-019-52350-y

**Published:** 2019-11-04

**Authors:** Melissa Ferguson, Ilka Lorenzen-Schmidt, W. Glen Pyle

**Affiliations:** Department of Biomedical Sciences, University of Guelph, Guelph, ON N1G 2W1 Canada

**Keywords:** Cardiovascular biology, Cardiovascular biology

## Abstract

The health effects of the endocrine disruptor Bisphenol A (BPA) led to its partial replacement with Bisphenol S (BPS) in several products including food containers, toys, and thermal paper receipts. The acute effects of BPS on myocardial contractility are unknown. We perfused mouse hearts from both sexes for 15 min with physiologically relevant doses of BPS or BPA. In females BPS (1 nM) decreased left ventricular systolic pressure by 5 min, whereas BPA (1 nM) effects were delayed to 10 min. BPS effects in male mice were attenuated. In both sexes ER-β antagonism abolished the effects of BPS. Cardiac myofilament function was not impacted by BPS or BPA in either sex, although there were sex-dependent differences in troponin I phosphorylation. BPS increased phospholamban phosphorylation at S16 only in female hearts, whereas BPA reduced phosphorylation in both sexes. BPA decreased phospholamban phosphorylation at T17 in both sexes while BPS caused dephosphorylation only in females. This is the first study to compare sex differences in the acute myocardial response to physiologically relevant levels of BPS and BPA, and demonstrates a rapid ability of both to depress heart function. This study raises concerns about the safety of BPS as a replacement for BPA.

## Introduction

Bisphenol A (BPA) is a ubiquitous monomer used in the manufacture of polycarbonates, and is found in a variety of consumer goods including food containers, baby bottles, nail polish, and food can linings, as well as industrial water pipes and dental sealants^1,2^. BPA is generally not considered a persistent chemical, with a half-life of approximately 6 h^3,4^ and no evidence of accumulation in humans after isolated doses. However, the continuous exposure by virtue of its almost ubiquitous occurrence in a variety of sources leads to a consistent presence in the body: BPA levels are detectable in over 90% of people in a wide range of populations^5^. Recent studies reported negative health effects of BPA exposure and led to successful campaigns to reduce human exposure^6,7^. Interestingly, despite the relatively new awareness of the negative effects of bisphenols, the estrogenic effects of these compounds were first described 80 years ago^8^.

The cardiovascular system has been the subject of investigation with respect to the negative health effects of BPA. Most studies found that BPA is associated with a higher risk of cardiovascular disease including coronary heart disease, angina, peripheral artery disease, and myocardial infarctions^9–11^. A study by LaKind *et al*.^12^ questioned the veracity of these claims, but this study itself was challenged by Posnack *et al*.^13^ who suggested a conflict of interest in the form of chemical industry funding. In mouse models of myocardial infarctions several studies found chronic BPA exposure worsened outcomes^14–16^. Furthermore, laboratory animal studies consistently show acute^5,17^ and life-long^18,19^ exposure of BPA at doses within the ranges seen in human populations has pro-arrhythmic effects. Studies showing negative cardiovascular effects of BPA exposure are particularly concerning given the widespread inclusion of BPA in medical devices which increase the levels in patients who are already at greater risk for cardiovascular complications^20^.

Bisphenol S (BPS) is increasingly being used as a substitute for BPA despite similar leeching issues and estrogenic effects^21^. BPS is found in a number of commonly used consumer products including food and beverage containers, toys, and thermal paper receipts^22^. A study examining individuals from the United States and 7 Asian countries detected BPS in 81% of urine samples with an average concentration of 2.6 nM, suggesting widespread exposure^23^. Of significant concern is the finding that acute BPS exposure shows a similar pro-arrhythmic effect^21^ and impairment of post-myocardial infarction recovery^14^ as BPA. Chronic exposure of BPS to zebrafish larvae induce developmental deformities in a number of organs including the heart^24,25^. However, beyond the arrhythmogenic effects of BPS, its acute and direct impact on heart function is unknown. The first objective of this study was to determine if acute and physiologically relevant exposure of the heart to BPS alters cardiac contractility, and how these effects compared to BPA.

The acute cardiac effects of BPA and BPS have been primarily confined to examining rhythm disorders and electrophysiological changes. Some studies examined myocyte contractility and found that bisphenols generally decrease myocyte contracility^3,21,26^. Only one study explored the impact of BPA on left ventricular contractility and found that acute BPA treatment reduced myocardial pressure development^27^. Investigations largely determined that the pro-arrhythmogenic effects of bisphenol exposure are mediated through disruptions in intracellular calcium handling, possibly through estrogen receptor-β activation^3,5,17,21^. Alterations in intracellular calcium handling can significantly impact myocardial contractility, given that calcium acts as a trigger for muscle contraction. However, whether cardiac myofilaments, the other half of the contractility equation, are affected by bisphenols has never been examined. The second objective of this study was to determine how acute BPA and BPS exposure impacts cardiac myofilament function.

## Results

### Acute exposure to BPA or BPS depresses left ventricular contractility through estrogen receptor-β

**Figure 1:**
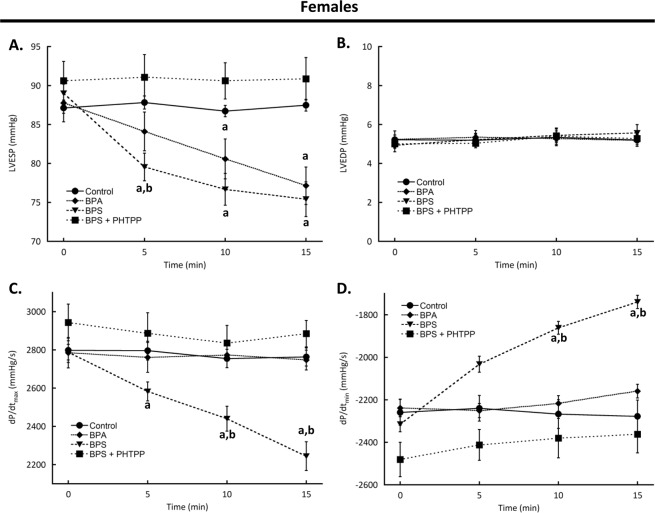
Acute exposure to bisphenol A (BPA) or S (BPS) rapidly decreases left ventricular function in female mice. Hearts excised from female CD1 mice were perfused with BPA (1 nM), BPS (1 nM), BPS + PHTPP (1 μM), or vehicle (0.0001% ethanol, Control) using a Langendorff apparatus. Functional parameters measured were (**A**) LVESP; (**B)** LVEDP; (**C**) Maximum rate of contraction; and (**D)** Rate of relaxation. **Key:** LVESP, left ventricular end systolic pressure; LVEDP, left ventricular end diastolic pressure; dP/dt_max_, maximum rate of contraction; dP/dt_min_, maximum rate of relaxation; PHTPP, estrogen receptor-β anatagonist. N = 10 for all groups except N = 6 for BPS + PHTPP. Values presented are mean ± SEM. ^a^p < 0.05 vs baseline of same group; ^b^p < 0.05 vs BPA.

Baseline left ventricular function values of female mice were not different between any treatment group (Table 1, all parameters p > 0.05). Perfusion of hearts with BPA decreased left ventricular end systolic pressure at 10 min (p = 0.0109) and was down by 12.1% (p = 0.0001) at 15 min compared to baseline levels. BPA had no significant effects on any other functional parameter assessed (Fig. 1, p > 0.05). The depressant effects of BPS occurred more quickly than those of BPA as left ventricular end systolic pressure was significantly reduced at 5 min (p < 0.0001) as compared to baseline levels. This decrease was significantly different than BPA treatment (p = 0.0024), and by 15 min had decreased by 15.3% (Fig. 1, p < 0.0001 vs baseline). Moreover, BPS decreased dP/dt_max_ by 5 min of treatment (p = 0.0274 vs baseline) and remained depressed at 15 min (p < 0.0001 vs baseline). BPS-dependent decreases in dP/dt_min_ were significant at 10 min (p = 0.0012) and remained below baseline at 15 min (p < 0.0001).

**Table 1:** Baseline left ventricular function for female mice.

Treatment	LVEDP (mmHg)	LVESP (mmHg)	dP/dt_max_ (mmHg/s)	dP/dt_min_ (mmHg/s)
Control	5.22 ± 0.24	87.13 ± 0.69	2798 ± 64	−2259 ± 60
BPA	5.24 ± 0.43	87.76 ± 2.42	2785 ± 79	−2238 ± 42
BPS	4.93 ± 0.33	89.00 ± 1.37	2788 ± 41	−2314 ± 36
BPS + PHTTP	5.01 ± 0.23	90.61 ± 2.49	2944 ± 96	−2481 ± 81

**Key:** LVEDP, left ventricular end diastolic pressure; LVESP, left ventricular end systolic pressure; dP/dt_max_, maximum rate of contraction; dP/dt_min_, maximum rate of relaxation; BPA, bisphenol A (1 nM); BPS, bisphenol S (1 nM); PHTPP, estrogen receptor-β anatagonist (1 μM). N = 10 for all groups except N = 6 for BPS + PHTPP. Values presented are mean ± SEM.

BPA-dependent changes in myocyte contractility are dependent on estrogen receptor-β activation^26^. To determine if the effects of BPS are mediated through a similar estrogen receptor-β-dependent pathway, we blocked receptor activation by pre-treating hearts with PHTPP. In hearts from female mice the functional depression that was observed with BPS treatment alone was abolished by estrogen receptor-β antagonism (Fig. 1, p > 0.05). PHTTP by itself had no impact on any measured parameter at any time (p > 0.05 vs baseline) (Supplementary Fig. 1).

### Effects of BPA and BPS on myocardial function are sex-dependent

**Figure 2:**
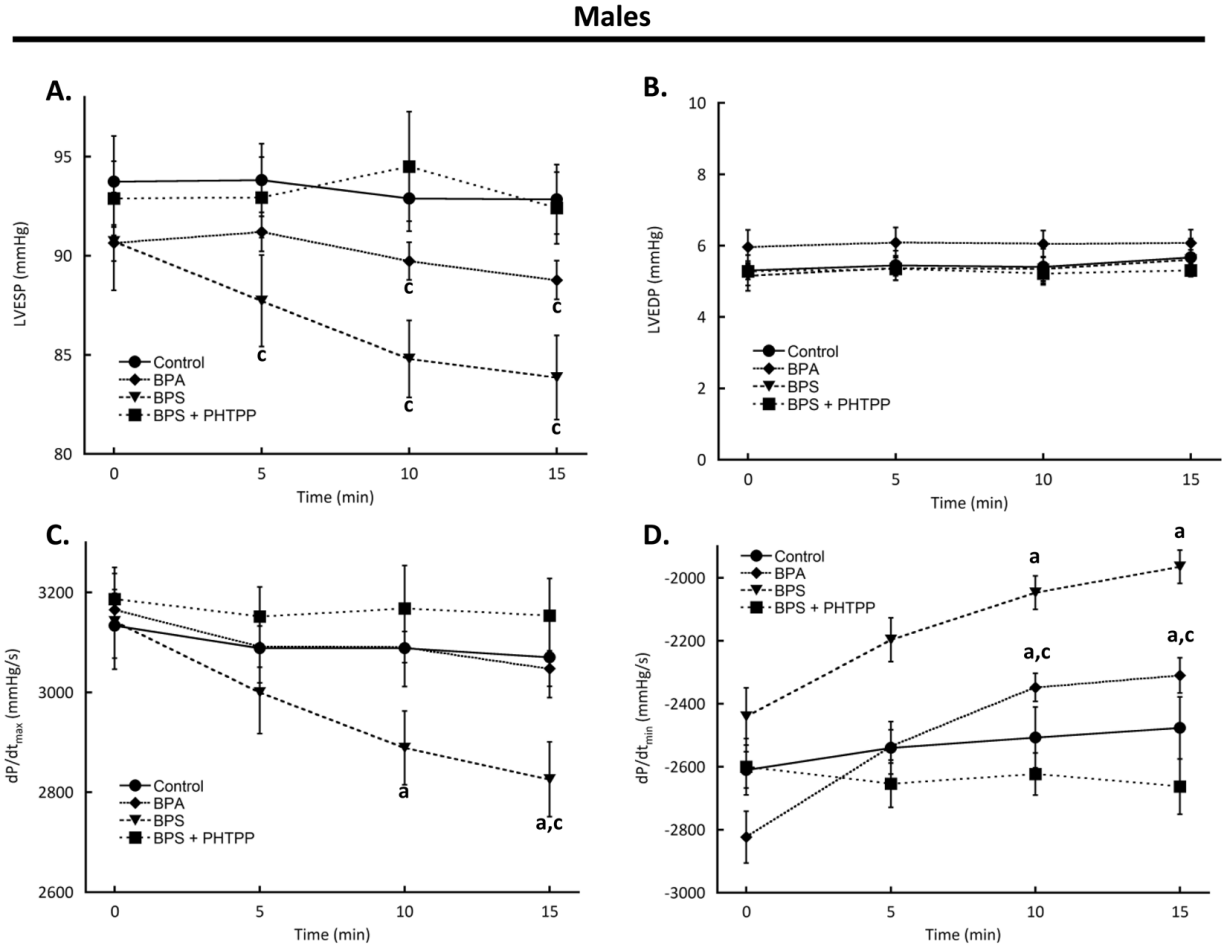
Bisphenol A (BPA) or S (BPS) rapidly decreases left ventricular function in male mice. Hearts excised from male CD1 mice were perfused with BPA (1 nM), BPS (1 nM), BPS + PHTPP (1 μM), or vehicle (0.0001% ethanol, Control) using a Langendorff apparatus. Functional parameters measured were (**A**) LVESP; (**B**) LVEDP; (**C**) Maximum rate of contraction; and (**D**) Rate of relaxation. **Key:** LVESP, left ventricular end systolic pressure; LVEDP, left ventricular end diastolic pressure; dP/dt_max_, maximum rate of contraction; dP/dt_min_, maximum rate of relaxation; PHTPP, estrogen receptor-β anatagonist. N = 10 for all groups except N = 6 for BPS + PHTPP. Values presented are mean ± SEM. ^a^p < 0.05 vs baseline of same group; ^b^p < 0.05 vs BPA; ^c^p < 0.05 vs same treatment and time in females.

Hearts from male mice were perfused with BPA or BPS using the same protocol as was employed for female mouse hearts. Baseline left ventricular function values of male mice were not different between any treatment group (Table 2, all parameters p > 0.05). Unlike hearts from female mice, the functional effects of BPA were confined to a decrease in dP/dt_min_ which was significantly slowed at 10 min (p = 0.0012) and was 18.2% lower by 15 min (p = 0.0022) (Fig. 2). The effects of BPS were similarly confined to contractility rates as dP/dt_min_ was significantly reduced starting at 10 min (p = 0.0376) and remained depressed at 15 min (p = 0.0051)/dP/dt_max_ declined following BPS treatment starting at 10 min (p = 0.0288) and remained below baseline by 10.1% after 15 min (p = 0.0085) (Fig. 2).

**Table 2:** Baseline left ventricular function values for male mice.

Treatment	LVEDP (mmHg)	LVESP (mmHg)	dP/dt_max_ (mmHg/s)	dP/dt_min_ (mmHg/s)
Control	5.31 ± 0.43	93.74 ± 2.29	3134 ± 66	−2610 ± 58
BPA	5.96 ± 0.48	90.64 ± 0.91	3166 ± 40	−2823 ± 82
BPS	5.15 ± 0.41	90.72 ± 2.47	3142 ± 96	−2440 ± 91
BPS + PHTTP	5.28 ± 0.23	92.89 ± 1.87	3187 ± 63	−2600 ± 89

**Key:** LVEDP, left ventricular end diastolic pressure; LVESP, left ventricular end systolic pressure; dP/dt_max_, maximum rate of contraction; dP/dt_min_, maximum rate of relaxation; BPA, bisphenol A (1 nM); BPS, bisphenol S (1 nM); PHTPP, estrogen receptor-β anatagonist (1 μM). N = 10 for all groups except N = 6 for BPS + PHTPP. Values presented are mean ± SEM.

Compared to hearts from female mice, hearts from male mice responded differently to both BPA and BPS treatment. The BPS-dependent decrease in LVESP was significantly less in male samples at all times (5 min p = 0.0005; 10 min p = 0.0094; 15 min p = 0.0151 vs females at same timepoints). The BPA impact on LVESP in males was attenuated at 10 min (p = 0.0277) and 15 min (p = 0.0012) compared to females. At 15 min BPS had a significantly reduced effect on dP/dt_max_ (p = 0.0169) in males as compared to females, whereas the effect of BPA on dP/dt_min_ was blunted in male samples at both 10 and 15 min (p < 0.0001 for both times) compared to females at the same times.

Although the effects of BPS on hearts from male mice were less compared to those in females, PHTPP had a similarly antagonistic effect (Fig. 2). PHTPP treatment alone had no effect (Supplementary Fig. 2).

### Cardiac myofilament function is not altered by BPA or BPS

**Figure 3:**
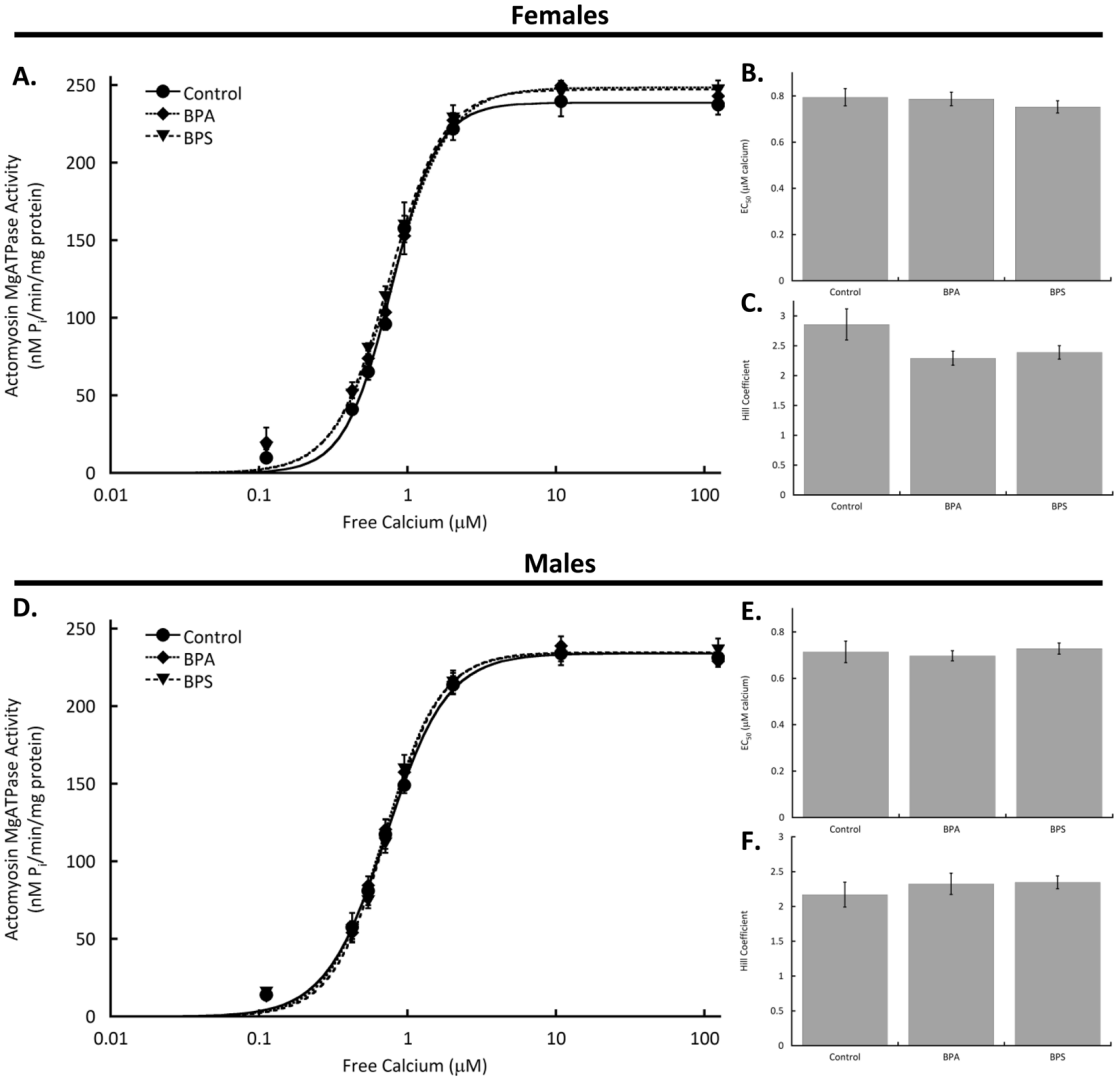
Cardiac myofilament function is not affected by bisphenol A (BPA) or S (BPS) in mice of either sex. Hearts excised from male and female CD1 mice were perfused with BPA (1 nM), BPS (1 nM), BPS + PHTPP (1 μM), or vehicle (0.0001% ethanol, Control) using a Langendorff apparatus. Cardiac myofilaments were isolated and function assessed using an actomyosin MgATPase assay. (**A**) Actomyosin MgATPase-calcium curves; (**B**) Actomyosin MgATPase EC_50_ values after; and (**C**) Hill coefficients of actomyosin MgATPase-calcium curves with BPA or BPS treatment of hearts from female mice. (**D**) Cardiac actomyosin MgATPase-calcium relationships; (**E**) Actomyosin MgATPase EC_50_ values; and (**F**). Hill coefficient of myofilaments isolated from hearts of male mice following BPA or BPS treatment. N = 10 for all groups. Values presented are mean ± SEM.

After perfusing hearts for 15 min with BPA or BPS, myocardial samples were used to isolate cardiac myofilaments. Myofilament function was assessed using a modified Carter assay to measure actomyosin MgATPase activity. Actomyosin MgATPase activity as represented by maximum activity, EC_50_, or Hill coefficient was not affected by BPA or BPS in samples from females or males (Fig. 3).

### Cardiac myofilament phosphorylation changes following BPA treatment are sex-dependent

**Figure 4:**
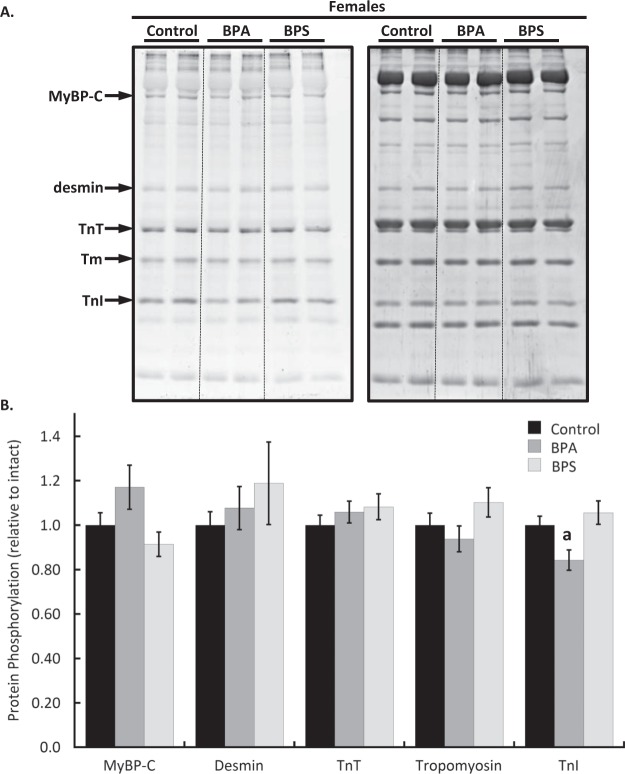
Cardiac myofilament phosphorylation changes in female mouse hearts following bisphenol A (BPA) or S (BPS) treatment. Hearts excised from female CD1 mice were perfused for 15 min with BPA (1 nM), BPS (1 nM), or vehicle (0.0001% ethanol, Control) and cardiac myofilaments were isolated. (**A**) Myofilament proteins were separated with 12% SDS-PAGE and stained with Pro-Q Diamond phosphoprotein stain (left) to detect phosphorylated proteins. Total protein load was measured by staining gels with coomassie stain (right). (**B**) Quantification of protein phosphorylation changes. **Key:** MyBP-C, myosin binding protein C; TnT, troponin T, Tm, tropomyosin; TnI, troponin I. N = 10 per group. Values presented are mean ± SEM. ^a^p < 0.05 vs baseline of same group; ^b^p < 0.05 vs BPA.

**Figure 5:**
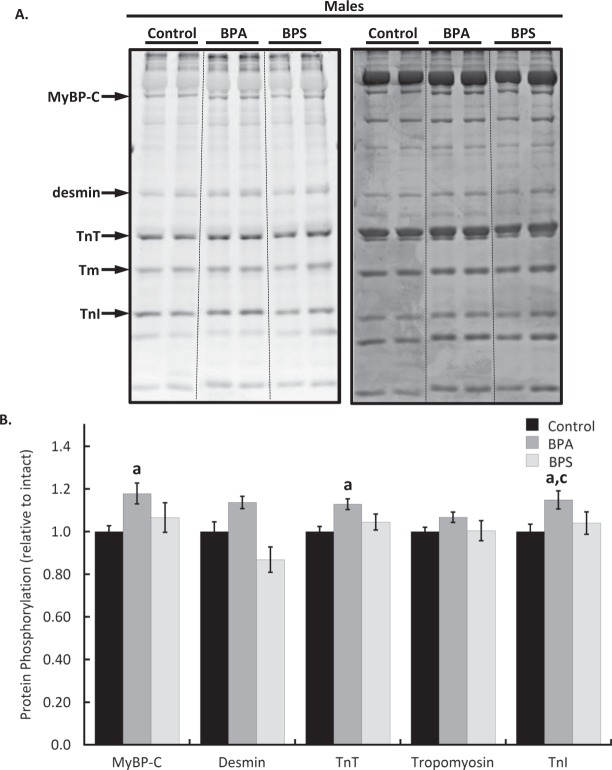
Bisphenol A (BPA) but not bisphenol S (BPS) alter cardiac myofilament phosphorylation in male mouse hearts. Hearts from male CD1 mice were perfused for 15 min with BPA (1 nM), BPS (1 nM), or vehicle (0.0001% ethanol, Control). (**A**) Isolated cardiac myofilament proteins were separated with 12% SDS-PAGE and phosphorylated proteins detected using Pro-Q Diamond phosphoprotein stain (left). Coomassie stain was used to determine total protein load (right). (**B**) Quantification of cardiac myofilament protein phosphorylation changes. Representative samples from the same gel are presented. **Key:** MyBP-C, myosin binding protein C; TnT, troponin T, Tm, tropomyosin; TnI, troponin I. N = 10 per group. Values presented are mean ± SEM. ^a^p < 0.05 vs baseline of same group; ^b^p < 0.05 vs BPA; ^c^p < 0.05 vs same treatment and time in females.

Cardiac myofilaments were isolated following 15 min of heart perfusion with BPA or BPS and resolved by SDS-PAGE. Total protein phosphorylation levels determined with Pro-Q Diamond phosphoprotein staining revealed that troponin I phosphorylation was reduced by 15.6 ± 4.6% (p = 0.0428) in hearts from female mice following BPA treatment (Fig. 4). In males, BPA increased the phosphorylation of myosin binding protein C (p = 0.0499), troponin T (p = 0.012), and troponin I (p = 0.0456) by 17.9 ± 4.9%, 12.8 ± 2.5%, and 14.9 ± 4.3% respectively (Fig. 5). Desmin phosphorylation increased by 13.7 ± 2.9% over control but it was not statistically significant (p = 0.107). BPS had no significant effect on the phosphorylation of any protein examined in either sex. The impact of BPA on troponin I phosphorylation was significantly different between sexes (p = 0.0001).

### Intracellular calcium handling is differentially altered by BPA and BPS in males and females

**Figure 6:**
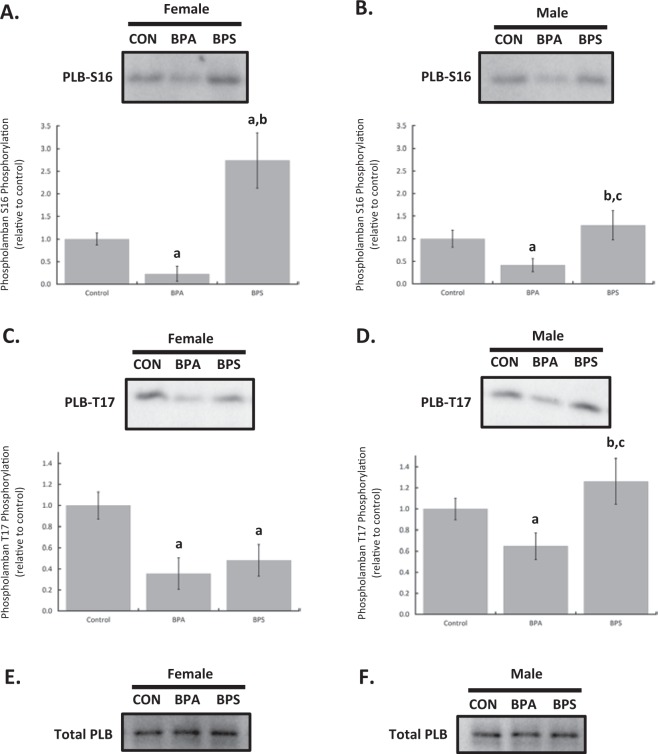
Bisphenol A (BPA) and bisphenol S (BPS) differentially affect phospholamban (PLB) phosphorylation in a sex-dependent manner. Hearts from female and male CD1 mice were perfused for 15 min with BPA (1 nM), BPS (1 nM), or vehicle (0.0001% ethanol, Control). Myocardial homogenates were probed with antibodies for phosphorylated phospholamban (PLB) at serine 16 (S16) or threonine 17 (T17). S16 phosphorylation in samples from female (**A**) and male (**B**) hearts. T17 phosphorylation in both sexes. (**C**,**D**) Total PLB levels in both sexes. (**E**,**F**) Representative samples from the same membranes are presented. N = 8 in each group for each sex. Values presented are mean ± SEM. ^a^p < 0.05 vs baseline of same group; ^b^p < 0.05 vs BPA; ^c^p < 0.05 vs same treatment and time in females.

Myocardial homogenates were resolved by SDS-PAGE, transferred to nitrocellulose, and probed with antibodies to detect phosphorylated phospholamban. Phosphorylated values were normalized to total phospholamban expression. Both female (p = 0.0139) and male (p = 0.0481) hearts perfused with BPA for 15 min had reduced phospholamban phosphorylation at serine 16. BPS increased serine 16 phosphorylation, in females (p < 0.0001). The BPS-dependent increase in phospholamban serine 16 phosphorylation in females was significantly different from males (p < 0.0001) who showed no significant change (p = 0.9965) (Fig. 6). Phosphorylation of phospholamban at threonine 17 was decreased with both BPA (p < 0.0001) and BPS (p = 0.0001) treatment in myocardial samples from female mice compared to controls. BPA also decreased threonine 17 phosphorylation in male samples (p = 0.0034) but BPS had no significant effect (p = 0.124). BPS-dependent effects on phospholamban threonine 17 phosphorylation was significantly different between males and females (p < 0.0001) while BPA was not (p = 0.1714).

## Discussion

The estrogenic and cardio-damaging effects of BPA led to its increasing replacement by BPS. Previous studies reported proarrhythmic effects of BPA^17,28^ that are absent following BPS exposure^21^, but the direct impact of BPS on whole heart function has never been examined. Our data show that a physiologically relevant concentration of BPS directly affects the heart and rapidly decreases myocardial contractility to a more significant degree than BPA in a manner that depends on sex. Inhibition of the cardio-depressant effects with an estrogen receptor-β antagonist implicates the involvement of an estrogen receptor-dependent pathway. Cardiac myofilaments, which have significant influence over myocardial performance, do not appear to be functionally impacted by either BPA or BPS, although both compounds altered the protein phosphorylation profile of the contractile apparatus. Phospholamban phosphorylation exhibited sex-dependent differences in response to BPA but not BPS, which is consistent with the sex-dependent differences in the myocardial response to the different bisphenols we found. In total this study is the first to identify rapid myocardial effects of BPS, to identify an estrogen receptor-β pathway as a mediator of the inhibitory effects of BPS on heart function, and to demonstrate sex-dependent differences in the myocardial response to acute BPS exposure.

In this study we show for the first time the rapid depressant effects of BPS treatment on isolated perfused mouse hearts. The inhibitory effects are largely mediated through a decrease in systolic pressure, as well as decreases in the rates of contraction (dP/dt_max_) and relaxation (dP/dt_min_). The effects of BPA were more modest as a significant decline in contractility was not seen until 10 min. Our observed effects of BPA are in slight disagreement with the work of Posnack and colleagues who did not see significant changes in pressure development in rat hearts until 100 nM BPA^27^. Posnack *et al*. looked at LVDP as a measurement of contractile force and did not present separate measurements for diastolic and systolic pressure. It appears from their figures that the decline in LVDP is driven primarily if not exclusively from systolic changes, which would be consistent with our results. It is unclear how long the effects took to occur in the earlier work by Posnack but we show rapid changes, which is the first time a study has specifically assessed the temporal nature of the effects of BPA or BPS on myocardial contractility. Furthermore, this study is the first to show that the acute effects of BPS on isolated mouse hearts are more potent than those of BPA. A decrease in cardiac contractility by BPS may lead to reduced heart function, including coronary hypoperfusion, increasing the risk of ischemic heart disease, as has been proposed for BPA^29^. The hypoperfusion of the heart in combination with a bisphenol-dependent stimulation of inflammation post-infarction^14,16^ may exacerbate the damage associated with ischemic heart disease. Given the trends towards replacing BPA with BPS in a number of consumer products, the results of our study are concerning.

Both our current study and that by Posnack *et al*.^27^ appear to contradict the BPA-dependent increase in cardiac myocyte contractility originally reported by Gao^3^. The discrepant effects between organ and cellular function may be attributed to the more complex environment of the organ system used in our current study which allows for both direct effects of bisphenols on cardiac myocytes, as well as indirect effects mediated through other cell types. Evidence for bisphenols stimulating the release of cardiomyocyte effectors by non-myocyte cell types was offered by Pant and colleagues who showed that the atrial effects of BPA are mediated through a NO-dependent mechanism^30^. In the heart there are several sources of NO including endothelial cells^31^. Chronic exposure to BPA decreases myocardial collagen and increases fibrosis, but the short treatment time in our study likely limits the impact of extracellular matrix remodeling on contractility^32–35^. Whether the effects of bisphenols on cardiac contractility are produced through direct stimulation of cardiac myocytes or indirect activation via non-myocyte cells should be explored in future studies.

Myofilament phosphorylation is a rapid and powerful mechanism to affect myocardial function. Relatively small changes in phosphorylation levels (eg. <10%) can profoundly influence contractility and susceptibility to pathological stressors^36^. Among the well-known phosphorylatable proteins within the sarcomere are myosin binding protein C, desmin, troponin T, troponin I, and tropomyosin, each of which has numerous modifiable amino acids. The functional effects of phosphorylation are complex and not straight-forward as the phosphorylation of multiple residues does not necessarily yield a change that is the sum of the individual modifications^37,38^. Our study shows that BPA and BPS both have modest but rapid effects on myofilament phosphorylation that differ between the sexes. Interestingly, these covalent modifications correspond with no detectable changes in myofilament function as assessed by an actomyosin MgATPase assay. Given that different phosphorylation sites (even within the same protein) can have contradictory effects on myofilament function, it is feasible that the phosphorylation changes discovered here offset each other to produce no net change in myofilament function. It is also possible that the functional impact of acute BPA and BPS exposure alters mechanical function independent of energy consumption. In dysfunctional or pathological states, cardiac myofilament energy consumption can be uncoupled from mechanical output, resulting in inefficient contraction^39^. Previous studies examining the cardiac effects of BPA and BPS have reported increased incidents of arrhythmias suggesting that these chemicals are pathologically stressful on the heart^10,29^. Our data clearly show that cardiac myofilaments are rapidly targeted by intracellular cascades activated by BPA and BPS treatment of the heart, but a more detailed examination of amino acids targeted by the intracellular pathways, along with a focused investigation of post-treatment myofilament mechanics is warranted.

Phospholamban can be phosphorylated at two sites *in vivo*: serine 16 by protein kinase A and threonine 17 by calcium-calmodulin-dependent protein kinase II^40^. Dephosphorylation does not appear to be dependent on site-specific phosphatases as type 1 protein phosphatase targets both amino acids^40^. Phosphorylation at either of these sites dissociates phospholamban from SERCA, boosting SERCA activity and increasing calcium removal. Functionally this leads to faster relaxation and enhanced myocardial contractility, but under certain conditions increased sarcoplasmic reticulum calcium can also be pro-arrhythmic^40,41^. Our study shows that treating hearts from female mice with BPS significantly increased phospholamban phosphorylation at serine 16 and decreased threonine 17 phosphorylation. Hearts of male mice treated with BPS showed no significant change in PLB phosphorylation, illustrating a sex-dependent difference. By contrast BPA significantly decreased phospholamban phosphorylation in hearts from both sexes to a similar degree. The effects of BPS have never been investigated before but are consistent with our novel finding that acute exposure decreases myocardial contractility. Furthermore, the lowered calcium loading that may result from decreased phospholamban phosphorylation is consistent with the work of Gao *et al*.^21^ who noted that BPS alone was not pro-arrhythmic. Our results, however, are not fully supportive of another study by Gao *et al*.^3^ who found that 15 min of BPA did not alter phospholamban phosphorylation, although there was a transient increase in threonine 17 phosphorylation. One potential reason for this apparent discrepancy is that Gao *et al*.^3^ treated isolated cardiac myocytes from female rats, whereas our study applied BPA to Langendorff perfused mouse hearts. The more complex biological system used in our study allows for effects of BPA to be mediated through direct targeting of cardiac myocytes, as well as indirect effects mediated through non-myocyte cells of the heart.

Interestingly the effects of BPA on PLB appear not to differ between the sexes whereas the impact on whole heart function exhibits some sex-dependent differences. By contrast BPS treatment more consistently showed sex-dependent differences across the assays used in this study. Our myofilament activity data failed to detect any sex-dependent differences in the response to BPA suggesting that the contractile apparatus is an unlikely contributor to the functional differences between female and male mice. Sex-dependent differences for BPS were confined to troponin I phosphorylation. Belcher *et al*.^26^ previously noted similar sex-dependent effects of BPA on ventricular myocytes and attributed the differences to interactions between estrogen receptor-α and -β. The intracellular mechanism for these effects was not identified by Belcher and colleagues although they did speculate that alterations in L-type calcium channel activity may play a role. To our knowledge there are no studies that have examined BPA-dependent changes to the intracellular environment such as pH which may also explain sex-dependent differences in the myocardial response to BPA. Sex-dependent differences in response to cardiac application of BPS were examined by Gao *et al*.^21^ who reported increased isoproterenol-triggered arrhythmias in hearts from female rats but not males. The mechanistic basis for the sex-dependent differences in arrhythmias were not conclusively determined although changes in PLB and ryanodine receptor phosphorylation were described. These potential mechanisms warrant further investigation to fully understand the role of sex in the heart’s response to bisphenols and their impact on cardiac function.

Our study is the first to demonstrate a role for estrogen receptor-β activation in altering whole heart function with BPS exposure. This finding is in agreement with Gao *et al*.^21^ who reported similar estrogen receptor-β-dependent effects on arrhythmias and calcium handling proteins, and Belcher *et al*.^26^ who implicated estrogen receptor-β in mediating the effects of BPA. Despite a consensus view that BPS and BPA activate estrogen receptor-β we demonstrate in this study that the impact of BPA and BPS on whole heart function is different. The ability of a single receptor type to mediate diverse cellular effects can be explained by the differential activation of intra-receptor switches^42^. Agonists that interact with membrane receptors through unique binding sites can activate an ensemble of switches that produce a structural conformation that activates distinct signaling pathways. Whether BPA and BPS interact with estrogen receptor-β at distinct sites or whether their binding induces unique structural changes in the receptor is beyond the scope of the current study.

The myocardial response to BPS and BPA differed by sex, as did the ability of these chemicals to alter the phosphorylation of myofilament and calcium handling proteins. Previous studies also reported sex-dependent differences that in general saw females respond more robustly to acute bisphenol exposure than males^17,21,26^. Similarly, Patel and colleagues found sex-dependent differences in cardiac structure and function responses to lifelong BPA exposure^43^. Estrogen receptor-β levels and distribution do not differ by sex in murine hearts^44^ making receptor expression differences an unlikely reason for the sex-dependent differences we observed. However, estrogen receptors can be phosphorylated to modify their response to agonists, and a recent study found that sex differences in estrogen receptor-β phosphorylation in rat cardiac fibroblasts may underlie differences in collagen regulation^45^. Whether sex-dependent differences in estrogen receptor-β phosphorylation contribute to the differences in the myocardial response to BPA and BPS is not known.

Bisphenols have wide ranging effects on several organ systems. Understanding the mechanisms by which they mediate effects at a systemic level requires a reductionist approach to identify local changes. For example, in the cardiovascular system BPA rapidly inhibits L-type Ca2+ channel opening in smooth muscle cells to induce vasodilation^46^. Changes in vascular resistance alters cardiac function, which interferes with an understanding of the direct effects bisphenols have on the heart. The doses we chose for this study are within the physiological range identified in human populations which supports the relevance of the study in terms of bisphenol levels. The choice to focus on isolated hearts does not capture the complexity of the body’s response to bisphenols, but it does facilitate an understanding of the impact of these compounds on heart function, and it provides novel insight into the mechanisms by which the effects are produced in the heart itself. Furthermore, the use of Langendorff perfused hearts allows for a more precise control of timing and dose of bisphenols, which decreases the variability inherent in orally dosed animals. This approach may not fully capture the experience and complexity of humans who are exposed to variable doses over long periods of time, but it does allow for a focused investigation into the mechanisms by which BPA and BPS impact the heart.

The negative effects of BPA on the cardiovascular system led to efforts to replace this agent with BPS. In this study we show rapid and profound depressant effects of BPS on heart function in mice of both sexes. Interestingly, the effects of BPS are more rapid and potent than those of BPA. The relatively recent move to replace BPA with BPS means that studies examining the impact of BPS on the heart are limited. The results of our study raise concerns over the effects of BPS on cardiovascular function and underscore the need for further investigation into the impact of BPS on cardiovascular health and physiology.

## Materials and Methods

### Animals

Female and male CD1 mice aged 78–105 days were purchased from Charles River Laboratories (St. Constant, QC, Canada). Mice were group housed at the Central Animal Facility at the University of Guelph on a 12 h light/dark cycle and provided food and water ad libitum. Animals were acclimated in the animal facility for at least one week prior to the initiation of treatment. All procedures were approved by and conducted in accordance with the guidelines set by the Animal Care and Use Committee of the University of Guelph and the Canadian Council on Animal Care.

### Bisphenol contamination

Animal housing equipment including cages and water bottles were verified to be BPS-free and not subject to BPA leach-out when exposed to standard washing and autoclaves processes. Plastic tubing used in Langendorff perfusion experiments are a bio-based formulation and contain no BPA or phthalates. All reagents were prepared using Type I UltraPure Water (Aqua Solutions, Jasper, GA).

### Langendorff perfusion

Langendorff perfused hearts allow for a focused investigation of the effects of bisphenols on the heart itself and permit more precise control of the timing and dose of treatments. Mouse hearts were perfused using a Langendorff apparatus as we have described previously^47^. Briefly, hearts were perfused for 15 min to establish baseline function. Baseline values were taken as an average of the last 30 seconds before treatment. Hearts were randomly treated for 15 min with BPA (1 nM), BPS (1 nM), or vehicle (0.0001% ethanol; control). Perfusion for 15 min was chosen to examine acute, nongenomic changes. Doses of BPA and BPS are similar to circulating levels found in humans^48,49^ and have been used by other studies^13,27^. Estrogen receptor-β antagonism was done by perfusing hearts for 10 min with 2-phenyl-3-(4-hydroxyphenyl)-5,7-bis(trifluoromethyl)-pyrazolo[1,5-a]pyrimidine (PHTPP, 1 μM) prior to and during agonist exposure^50^. PHTPP is a silent estrogen receptor-β antagonist that is 36-fold selective over estrogen receptor-α^50^. All chemicals were purchased from MilliporeSigma (Oakville, ON, Canada). After perfusion hearts were snap frozen in liquid nitrogen and stored at −80 °C for molecular analysis.

### Myofilament isolation

Cardiac myofilaments were isolated using our published protocol^47^. All steps were conducted at 4 °C. Hearts were homogenized with a hand held homogenizer in Standard Buffer (60 mM KCl, 30 mM imidazole (pH 7.0), 2 mM MgCl_2_) containing protease and phosphatase inhibitors. The homogenate was centrifuged for 15 min at 12,000 g and the pellet resuspended in Standard Buffer plus 1% Triton X-100. The homogenate was gently mixed for 45 min and was then centrifuged at 1,100 g for 15 min. The pellet was washed with Standard Buffer and spun at 1,100 g for 15 min three times. Protein concentration was measured with a Bio-Rad Bradford Protein Assay (Bio-Rad Laboratories Ltd., Mississauga, ON, Canada).

### Actomyosin MgATPase assay

Actomyosin MgATPase activity was measured using a modified Carter assay^47^. Isolated cardiac myofilaments (25 μg) were incubated in reaction buffers containing varying levels of free calcium. Free calcium was calculated using the program described in Bers *et al*.^51^. Buffers containing free calcium concentrations of pCa 4.0 (activating) and pCa 9.0 (relaxing) were mixed to prepare reaction buffers. Myofilaments were incubated in reaction buffers for 10 min at 32 °C and the reactions quenched with ice cold 10% trichloroacetic acid. The production of inorganic phosphate was measured by adding equal volumes of 0.5% FeSO_4_ and 0.5% ammonium molybdate in 0.5 M H_2_SO_4_.

### Myofilament protein phosphorylation

Total phosphorylation of myofilament proteins was determined with Pro-Q Diamond phosphoprotein stain (Thermo Fisher Scientific, Mississauga, ON, Canada) according to the manufacturer’s instructions as we have done previously. Myofilament proteins (10 μg) were separated with 12% SDS-PAGE and fixed in 50% methanol-10% acetic acid. Gels were stained with Pro-Q Diamond phosphoprotein stain for 2 h, followed by 3 h of destaining (20% acetonitrile, 50 mM sodium acetate, pH 4.0). Gels were imaged using a Bio-Rad ChemiDoc MP Imaging System (Bio-Rad Laboratories Ltd., Mississauga, ON, Canada) and data were analysed with ImageJ (NIH, Bethesda, MD, USA). Total protein load was determined by Coomassie staining of the same gels and protein phosphorylation was normalized to total protein.

### Immunoblotting

Myocardial samples were homogenized and separated using 16% SDS-PAGE, and then transferred (300 mA, 50 min, room temperature) to nitrocellulose membranes. Membranes were blocked with 5% dry milk powder in TBS (1 h, room temperature) and then probed overnight at 4 °C with antibodies against phospholamban phosphorylated at serine 16 (PLB-S16, 1:2000, sc-12963), threonine 17 (PLB-T17, 1:2000, sc-17024-R), or total phospholamban (Total PLB, 1:2000, sc-393990) (Santa Cruz Biotechnology, Inc., Dallas, TX, USA). Membranes were incubated (1 h, room temperature) with secondary antibodies (1:5000; goat anti-mouse, A2304, SigmaMillipore, Oakville, ON, Canada) conjugated to horse-radish peroxidase (. Bands were detected using Western Lightning ECL Pro (PerkinElmer Inc., Watham, MA, USA) and a Bio-Rad ChemiDoc MP imaging system. Densitometric analysis was performed using Image J (NIH, Bethesda, MD, USA). Phosphorylated phospholamban was normalized to total phospholamban levels.

### Statistical analysis

All data are shown as mean ± SEM. Statistical analysis was done using two-way ANOVA and a post-hoc Tukey’s Test. p < 0.05 was considered statistically significant.

### Datasets

No datasets were generated or analysed in this current study.

## Supplementary information

Supplementary Figures
