# Supplementary material for: Bisphenol S rapidly depresses heart function through estrogen receptor-β and decreases phospholamban phosphorylation in a sex-dependent manner
Source: Sci Rep. 2019 Nov 4;9:15948. doi: 10.1038/s41598-019-52350-y (PMC6828810; doi:10.1038/s41598-019-52350-y)
Supplement: Supplementary file 1 — Supplementary Figures [file 41598_2019_52350_MOESM1_ESM.pdf]

Bisphenol S rapidly depresses heart function through estrogen receptor-β and decreases phospholamban phosphorylation in a sex-dependent manner

Melissa Ferguson (mferg56@bu.edu), Ilka Lorenzen-Schmidt (ilorenzen-schmidt@ottawaheart.ca), and W. Glen Pyle (gpyle@uoguelph.ca)
Department of Biomedical Sciences, University of Guelph, Guelph, ON N1G 2W1

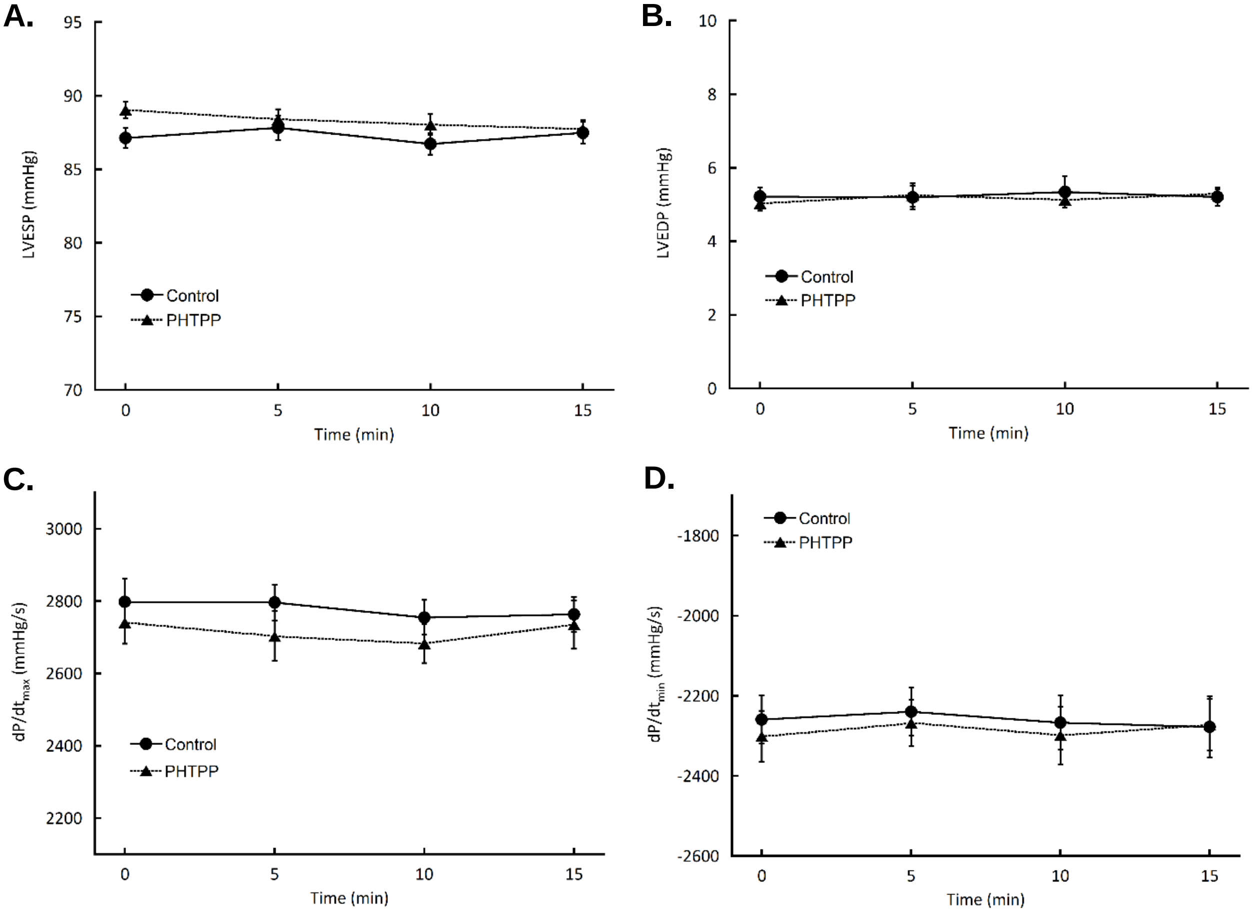

Supplementary Figure 1. ***Acute exposure to the estrogen receptor-β antagonist PHTPP does not impact left ventricular function in female mice.*** Hearts excised from female CD1 mice were perfused with PHTPP (1 μM; N=5) or vehicle (0.0001% ethanol, Control; N=10) using a Langendorff apparatus. Functional parameters measured were **A.** LVESP; **B.** LVEDP; **C.** Maximum rate of contraction; and **D.** Rate of relaxation . **Key:** LVESP, left ventricular end systolic pressure; LVEDP, left ventricular end diastolic pressure; dP/dt_{max}, maximum rate of contraction; dP/dt_{min}, maximum rate of relaxation; PHTPP, estrogen receptor-β anatagonist. Values presented are mean ± SEM. Control values are same as presented in Figure 1.

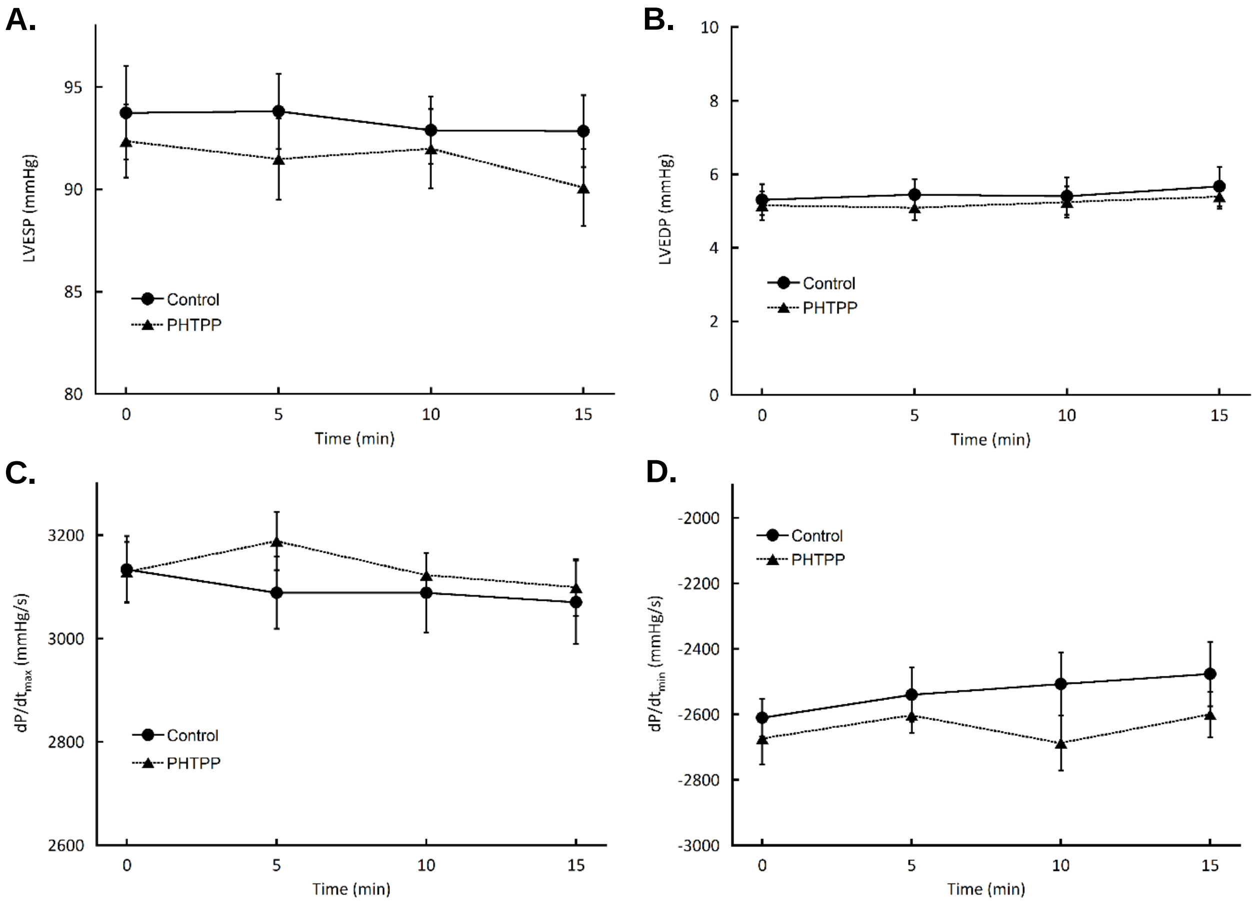

Supplementary Figure 2. ***Acute exposure to the estrogen receptor-β antagonist PHTPP does not impact left ventricular function in male mice.*** Hearts excised from female CD1 mice were perfused with PHTPP (1 μM; N=5) or vehicle (0.0001% ethanol, Control; N=10) using a Langendorff apparatus. Functional parameters measured were **A.** LVESP; **B.** LVEDP; **C.** Maximum rate of contraction; and **D.** Rate of relaxation . **Key:** LVESP, left ventricular end systolic pressure; LVEDP, left ventricular end diastolic pressure; dP/dt_{max}, maximum rate of contraction; dP/dt_{min}, maximum rate of relaxation; PHTPP, estrogen receptor-β anatagonist. Values presented are mean ± SEM. Control values are same as presented in Figure 2.

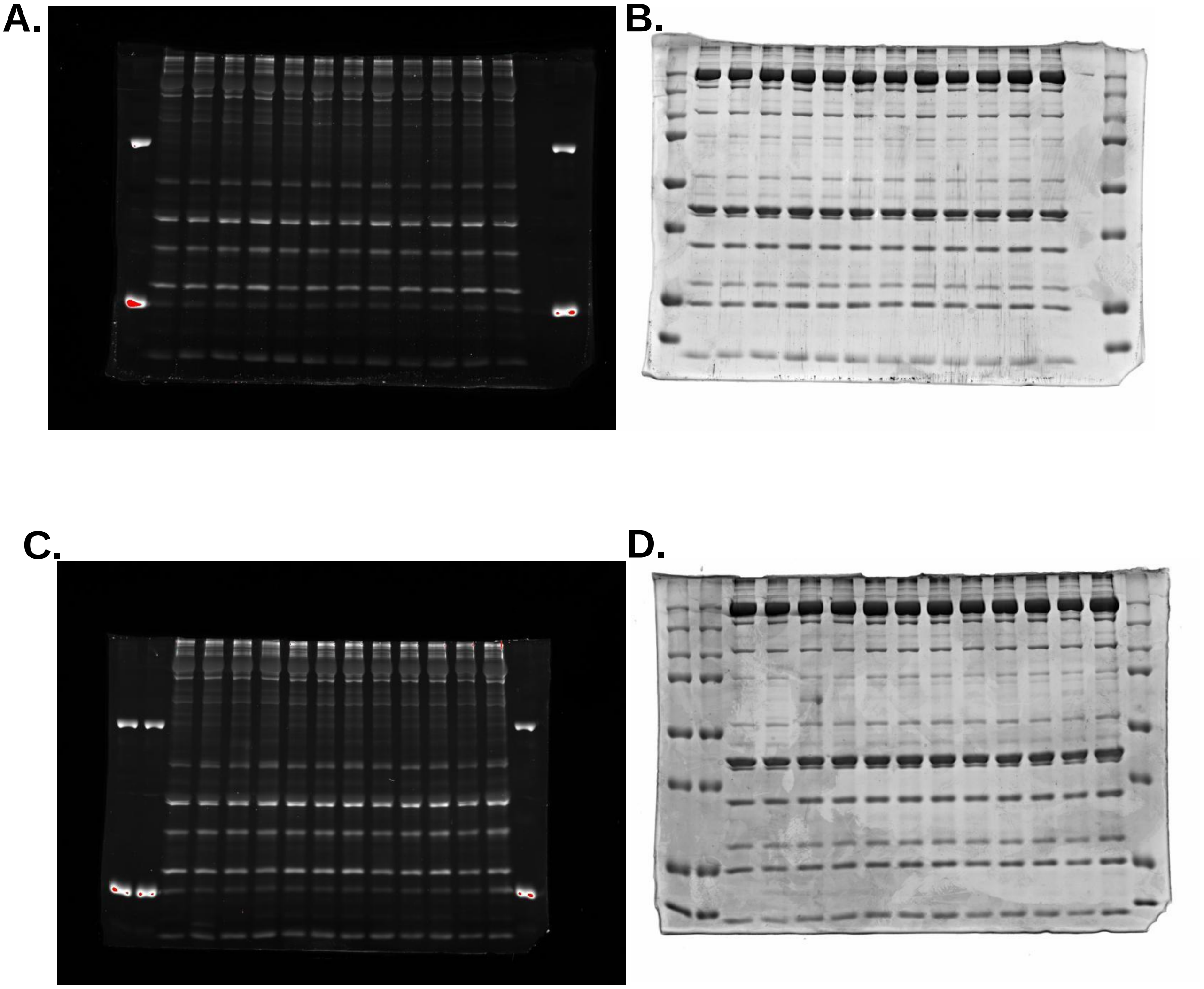

Supplementary Figure 3. A. Figure 4 ProQ phosphorylation gel. **B.** Coomassie stained gel. **C.** Figure 5 ProQ phosphorylation gel. **D.** Coomassie stained gel.

A.

B.

C.

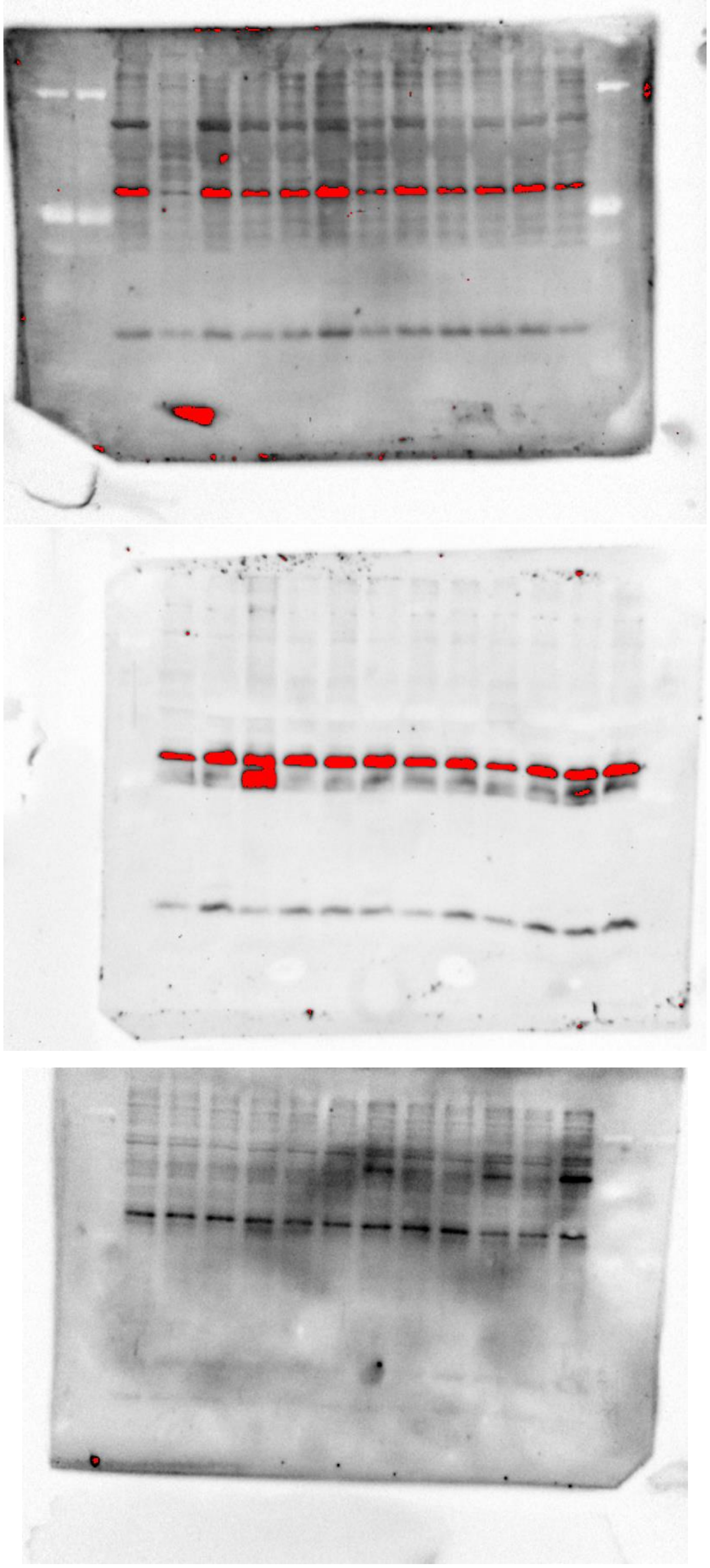

Supplementary Figure 4. A. Figure 6A and B. **B.** Figure 6C and D. **C.** Figure 6E and F.
